# Developing future heat-resilient vegetable crops

**DOI:** 10.1007/s10142-023-00967-8

**Published:** 2023-01-24

**Authors:** Faisal Saeed, Usman Khalid Chaudhry, Ali Raza, Sidra Charagh, Allah Bakhsh, Abhishek Bohra, Sumbul Ali, Annapurna Chitikineni, Yasir Saeed, Richard G. F. Visser, Kadambot H. M. Siddique, Rajeev K. Varshney

**Affiliations:** 1 Department of Agricultural Genetic Engineering, Faculty of Agricultural Sciences and Technologies, Nigde Omer Halisdemir University, 51240 Nigde, Turkey; 2 College of Agriculture, Oil Crops Research Institute, Fujian Agriculture and Forestry University (FAFU), Fuzhou, 350002 China; 3 State Key Laboratory of Rice Biology, China National Rice Research Institute, Chinese Academy of Agricultural Sciences (CAAS), Hangzhou, China; 4 Centre of Excellence in Molecular Biology, University of the Punjab, Lahore, Pakistan; 5 State Agricultural Biotechnology Centre, Centre for Crop and Food Innovation, Murdoch University, Murdoch, 6150 Australia; 6 Akhuwat Faisalabad Institute of Research Science and Technology, Faisalabad, Pakistan; 7 Center of Excellence in Genomics and Systems Biology, International Crops Research Institute for the Semi-Arid Tropics (ICRISAT), Hyderabad, India; 8 Department of Plant Pathology, Faculty of Agriculture, University of Agriculture, Faisalabad, 38040 Pakistan; 9 Plant Breeding, Wageningen University & Research, Droevendaalsesteeg 1, 6708 PB, 15 Wageningen, The Netherlands; 10 The UWA Institute of Agriculture, The University of Western Australia, Perth, 6001 Australia

**Keywords:** Abiotic stress, Biotechnology, Climate change, Heat stress, GWAS, Genome editing, QTL mapping

## Abstract

Climate change seriously impacts global agriculture, with rising temperatures directly affecting the yield. Vegetables are an essential part of daily human consumption and thus have importance among all agricultural crops. The human population is increasing daily, so there is a need for alternative ways which can be helpful in maximizing the harvestable yield of vegetables. The increase in temperature directly affects the plants’ biochemical and molecular processes; having a significant impact on quality and yield. Breeding for climate-resilient crops with good yields takes a long time and lots of breeding efforts. However, with the advent of new omics technologies, such as genomics, transcriptomics, proteomics, and metabolomics, the efficiency and efficacy of unearthing information on pathways associated with high-temperature stress resilience has improved in many of the vegetable crops. Besides omics, the use of genomics-assisted breeding and new breeding approaches such as gene editing and speed breeding allow creation of modern vegetable cultivars that are more resilient to high temperatures. Collectively, these approaches will shorten the time to create and release novel vegetable varieties to meet growing demands for productivity and quality. This review discusses the effects of heat stress on vegetables and highlights recent research with a focus on how omics and genome editing can produce temperature-resilient vegetables more efficiently and faster.

## Introduction

Plant parts that can be eaten as food are termed vegetables. These plant parts can be leaves, stems, tubers, roots, bulbs, and fruits (Radovich 2018). Vegetables belong to a diverse group of plants. More than 200 plants are reported as vegetables all around the world. Only 30–40 vegetable crops are commonly used for planting (Chen et al. 2019a). All vegetable crops which are grown outside their optimal temperature range (either too cold or too hot) are affected by stress. Due to global warming, a decrease of 41% in the production of vegetables was recorded from 1965 to 2016 (Scheelbeek et al. 2018). Due to current climate changes, environmental stresses cause disorders and affect the plant’s physical structure. For instance, heat stress (HS) affects the potatoes’ yield, and a 35% decrease in production was recorded under high temperature (Rykaczewska 2015).  Likewise, 93, 91, and 98% yield reduction was recorded in sweet pepper, chilli pepper, and tomatoes, respectively (Aleem et al. 2020). High temperature affects growth, poor fruit set and ultimately yield (Kawasaki and Yoneda 2019). There is an increase of 0.73 ℃ recorded in 100 years (Ito et al. 2022). Vegetables are important for a continuous food supply and are also affected by heat, whether day or night (Nie et al. 2022). In Chinese cabbage, high temperature affects flowering and leads towards early flowering (Huang et al. 2020). Other than flowering time, it also decreases seed production and flower number (Liu et al. 2012). High temperature also affects the quality and pigmentation of wild cabbage (Johansen et al. 2017). In tomato and potato, HS affects the fruit setting rate and production, respectively (Kim and Lee 2020; Cappetta et al. 2021). Many vegetable crops are, depending on their developmental stage and type of crop, susceptible to a variety of stresses.

Abiotic stresses such as temperature stress (cold and heat), salinity, and drought directly affect the yield and quality of plants (Bulgari et al. 2019; Raza et al. 2022a, b, c, d). Global warming is a dangerous concern for many vegetable crops because of the rising of optimal temperatures needed for growth, production and sustainable yields. Climate change not only results in higher but more erratic and fluctuating temperatures, sometimes too hot but even too cold. Depending on the type of vegetable crop and its origin, some can withstand freezing, prolonged cold or even heat (Bisbis et al. 2018; Raza et al. 2021b). Thus, there is a need for improved varieties of vegetables that can tolerate not only heat but also cold and in general temperature stress. Researchers are trying to understand the heat and cold response mechanisms which can help produce improved vegetable cultivars and strengthen the breeding program (Aleem et al. 2020; Raza et al. 2021b; Kang et al. 2022; Chaudhary et al. 2022). New genomic tools such as recombinant DNA technology, mutation breeding, and genome editing can help speed up the process of producing temperature-resilient crops (Aleem et al. 2020; Raza et al. 2021b; Varshney et al. 2020, 2021a, b; Yaqoob et al. 2023). Conventional breeding combined with genome editing can play a part in introducing/altering the functions of genes of interest in the crop and can produce transgene-free plants. The current review highlights the influence of HS on the productivity of major vegetable crops. Additionally, their morpho-physiological and biochemical responses to stress are discussed, and how vegetables can be made resilient against these extreme climate conditions by using different approaches.

## How does climate change give rise to temperature stress?

**Fig. 1:**
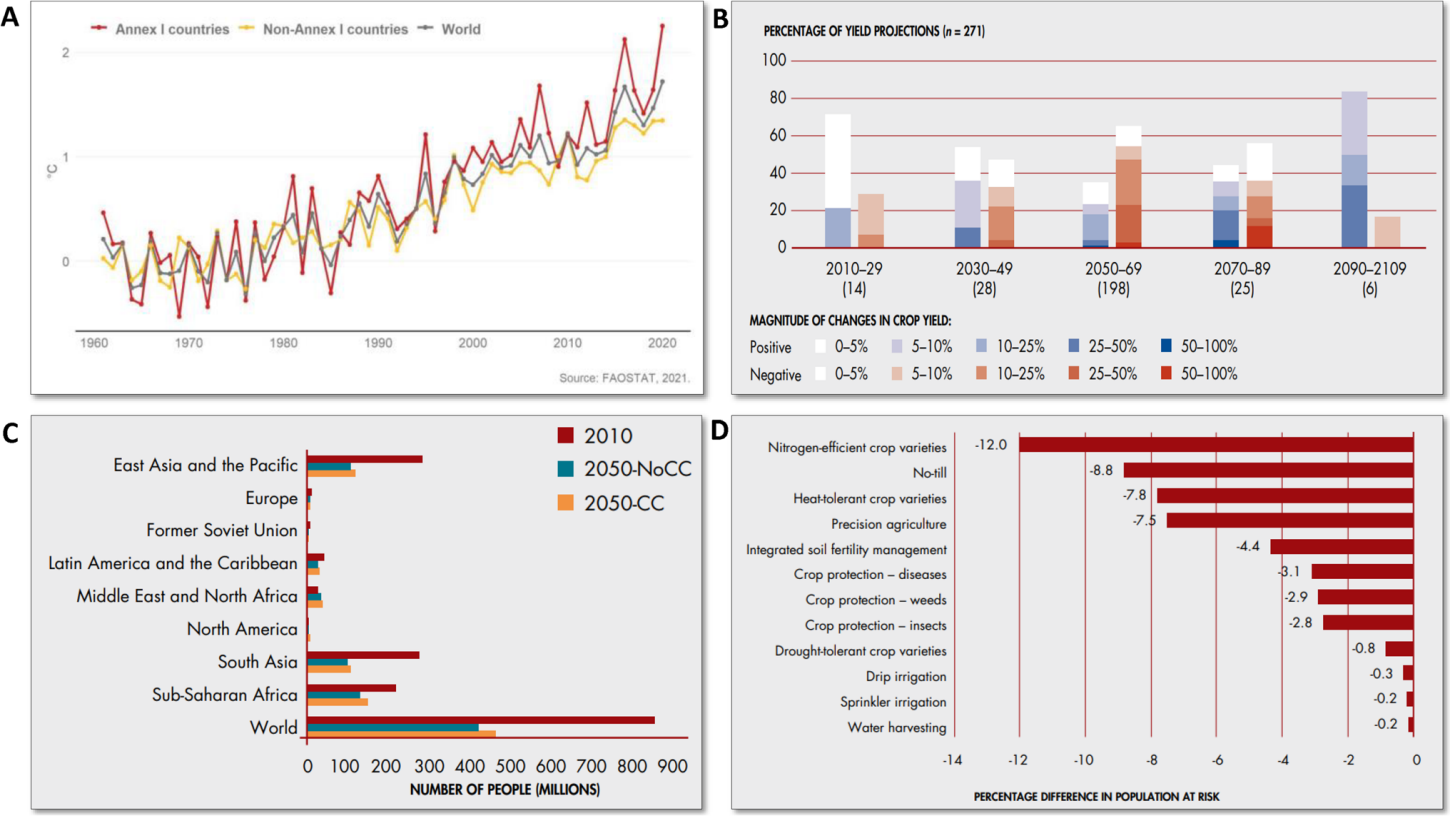
The impact of climate change on temperature variation and crop yields. **A** Mean annual temperature anomalies over land for World, Annex I countries (developed, according to the climate convention) and non-Annex I countries (developing). Source: https://www.fao.org/3/cb4410en/cb4410en.pdf. **B** Projected positive and negative changes in crop yields in developed regions owing to climate change. **C** The impacts of climate change on the population at risk of hunger in 2050, by region. **D** Change in 2050 in the number of people at risk of hunger, relative to the baseline scenario, after the adoption of improved agricultural technologies. Source: https://www.fao.org/3/i6030e/I6030E.pdf

Globally averaged surface temperature is increasing every year due to several factors. One of the key factors is altered anthropogenic activities that induce climatic changes at a rapid pace (Fig. 1) (Junaid et al. 2021). Change in the emission of atmospheric gases composition generates higher heat as it interferes with the flow of energy (Karimi et al. 2021). Therefore, disturbed greenhouse gas emissions due to changes in the use of agricultural land results in triggering global warming. Extreme climatic conditions frequently increase long-lasting heat waves. It is also expected to adversely affect the Earth’s vegetation patterns with a threat to global food security. For instance, climatic predictions suggest an increase in temperature, especially in the Mediterranean countries that will face hotter and drier weather conditions resulting in enormous yield losses (Morán-Ordóñez et al. 2020). Temperature is one of the essential factors that control the worldwide species distribution, and likewise, the general biological processes of plants are sensitive to changes in the climate. Climatic changes will cause large fluctuations in temperature leading to alterations in the thermal environment of the plants. Changes in temperature (cold and heat) mediated by climate change are further elaborated below. It is clear that these climatic changes will have an impact on agriculture and horticulture, including vegetable crops, in the broadest sense.

Water availability is decreasing yearly, which is an essential part of the healthy production of vegetables. Limited water availability for plants causes drought stress. During stress closure of stomata is closely linked with water use efficiency, a vital parameter of plant response when facing stress (Hatfield and Dold 2019). Under HS, it is expected that plants can increase the uptake of water for reproduction and growth (Grossiord et al. 2017). It is defined as deficient soil moisture conditions or below-normal precipitation due to harsh climatic conditions for a prolonged period of time that ultimately cause poor growth, yield, and quality of vegetables (Chaudhry et al. 2021). Additionally, climate change is another factor that results in lower rainfall patterns and higher temperatures. Therefore, drought triggers the negative influence of heat stress (Giordano et al. 2021). Generally, drought coincides with higher air temperature that enhances evapotranspiration resulting in closure of stomata, reduction in photosynthetic rate and damage to photosynthetic pigments (Giordano et al. 2021). Currently, several studies have been reported to reveal the effect of combined stresses to adapt vegetable crops to cope with future climatic changes to ensure food security (Demirel et al. 2020; Mushtaq et al. 2022; Gökçe et al. 2022).

### Heat stress

**Fig. 2:**
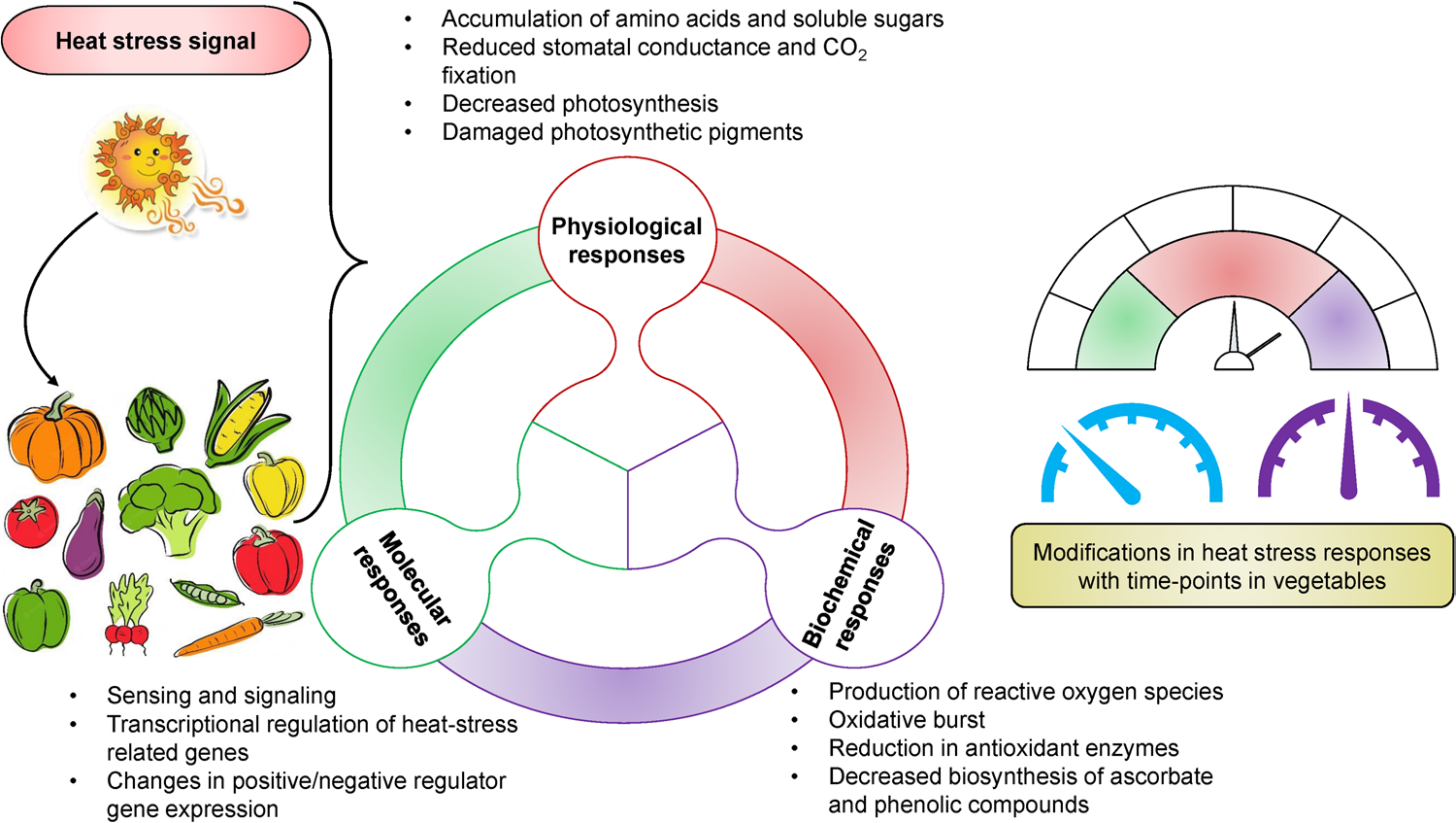
Vegetable crop responses to heat stress. Several responses occur at different levels, including biochemical, molecular, and physiological which affects the nutritional or production value of the vegetable. Further, these responses vary with the height of the stress conditions and time points on which they are afflicted

Heat stress (HS) can be defined as the increase in temperature for a specified period resulting in irreversible harm to the plants, which usually occurs with a temperature rise of 10–15 °C above the threshold level (Raza et al. 2022a; Zahra et al. 2021; Raza 2022). HS alters the impact of other abiotic stresses. Higher air temperature increases the transpiration rate, which demands higher water uptake by plants (Asim et al. 2021). It also increases the soil temperature which increases evaporation, and results in the shortage of available water for plants either for a shorter period or may result in prolonged drought stress (Hassan et al. 2021b). Additionally, a higher evaporation rate favors the increased accumulation of salts that triggers salinity stress conditions for the plants (Liu et al. 2021a). It can also cause an imbalanced nutrient supply, resulting in nutrient stress (Safdar et al. 2019). Whenever plants are exposed to HS, they manifest several modifications at the biochemical, physiological, and molecular stages, as described in Fig. 2. Therefore, it is essential to determine the severity of HS for a particular vegetable crop in the critical plant growth stage. Fluctuation in temperature is an environmental factor that negatively affects crop yield. Temperature’s physical effects include solar radiation, which can be measured based on plant’s heat balance (Raza et al. 2021b). Its physiological influence on plants consists of a reduction in stomatal conductance leading to poor photosynthetic rate and respiration that disturbs the plant growth. Worldwide, agriculture is one of the main sectors that are prone to climatic changes (Abbas 2013). HS is primarily responsible for influencing the growth and development of plants. Temperature requirement varies for the growth of each plant species. HS negatively affects plants’ growth and productivity, leading to changes in morpho-physiological and molecular responses (Asim et al. 2021).

Recently, frequent intense heat events caused several phenological disturbances among different plant species globally (Lamichaney et al. 2021). According to predicted climatic models, yield losses to crop plants due to HS may increase by 40% in the coming half of this century (Shahzad et al. 2021). HS increases plant’s vulnerability depending upon the sensitivity of plants, intensity, duration, and widespread severe heat events (Jagadish et al. 2021). It is expected that HS may result in 32% yield losses of potatoes by 2050 (Hijmans 2003). The HS at the early growth stage prevents seed germination and seedling emergence. As the plant enters the vegetative growth stage, they suffer from impaired photosynthetic pigments, perception of light, metabolism of carbon, organic solute translocations resulting in reduced growth (Hassan et al. 2021a; Karkute et al. 2021; Roeber et al. 2021). The physiological effect on vegetables caused by temperature extremes is shown in Table 1. HS can also halt the morphological growth of vegetables. It inhibits the cellular growth that limits the growth of leaves, stems, and branches of plants (Zahra et al. 2021). Additionally, prolonged exposure to HS restricts root growth, discoloration of fruit, and ultimately results in a poor or even no yield of vegetables (Kawasaki and Yoneda 2019). For instance, optimal potato tuber yield can be achieved at a temperature of 14–22 °C. The ideal tuber growth starts at 25 °C, and its vegetative growth reduces with an air temperature above 39 °C. Additionally, soil temperature above 18 °C combined with higher air temperature significantly reduces the tuber yield. HS also decreases the yield of the tuber by triggering the sprouting of the seed tubers (Aksoy et al. 2015, 2021; Demirel et al. 2020). Leafy vegetables that include cauliflower, cabbage, and broccoli are cool-season crops; therefore, HS during their growth period causes detrimental growth losses. In comparison, tomato and pepper are extremely sensitive to HS, especially during the reproductive phase. It inhibits fruit set. It suggests that HS affects the fertilization stage (Aleem et al. 2020; Saleem et al. 2021). Tomato flowering period is more prone to HS, which can cause impairment of pollen functions. HS accompanied by longer days initiates flowering of spinach and lettuce, which leads to a decline in the quality of the vegetables. HS kills the plants by disrupting the plant’s enzymatic activities, resulting in oxidative burst and damaged metabolism of the plant (Raza et al. 2021b; Raza 2022; Zahra et al. 2021). It is more lethal to plant growth as it also triggers secondary stresses.

**Table 1:** The effect of temperature stress on physiological attributes of some vegetables

Vegetable	Temperature	Effects	Reference
Pea	38 ℃	Reduced chlorophyll and carotenoid contents	Georgieva and Lichtenthaler (2006)
Potato	25 ℃	Reduce the rate of photosynthesis and carotenoid content	Aien et al. (2011)
Leafy radish	40 ℃	Changes in stomatal characteristics, and decreased photosynthetic rate	Chen et al. (2014)
Mungbean	40 ℃	Reduced relative water contents and damaged chlorophyll contents	Nahar et al. (2015)
Cauliflower	40 ℃	Decrease in chlorophyll fluorescence	Lin et al. (2015)
Cabbage	40 ℃	Reduce noted in photosynthesis activity	Chang et al. (2016)
Mungbean	40 ℃	Decrease the viability of pollen	Sharma et al. (2016)
Okra	45 ℃	Smaller sacs of pollen and affect the germination of pollen	Hayamanesh (2018)
Tomato	36 ℃	Decreased chlorophyll contents and CO_2_ assimilation rate	Zhou et al. (2017)
Pepper	40 ℃	Decreased chlorophyll contents	Haq et al. (2019)
Radish	40 ℃	Reduced chlorophyll contents	Yang et al. (2019)
Chickpea	40 ℃	Decrease in the content of chlorophyll	Kaloki et al. (2019)
Carrot	35–38 ℃	Decreased cell membrane stability, increased relative cell injury	Nijabat et al. (2020)
Water spinach	42 ℃	Decreased chlorophyll content, photosynthetic rate, carbon fixation, and increased respiration rate	Guo et al. (2020)
Sweet potato	37 ℃	Reduction in chlorophyll contents	Heider et al. (2021)
Cabbage	42 ℃	Decreased stomatal conductance rate, and chlorophyll contents	Moradpour et al. (2021)
Potato	39 ℃	Decreased gaseous exchange and damaged photosynthetic pigments	Şanlı and Öztürk Gökçe (2021)

Dry weather conditions cause water shortage with the immediate closure of stomata to restrict water loss. However, most plants have a reserve response that permits plants to survive HS conditions. It also disrupts the uptake of balanced mineral nutrition, damages the plant’s defensive system, triggers oxidative stress, and results in excessive production of reactive oxygen species (ROS) (Medina et al. 2021). The degree of tolerance of vegetables to HS with the changing temperature is crucial to estimate their heat tolerance levels to adapt them against future harsh temperature conditions.

## Heat stress-related physiological changes in major vegetables

Vegetables acquire several mechanisms to survive under HS conditions. The foremost mechanism is the maintenance of normal physiological functioning (Bisbis et al. 2018). HS first disrupts gaseous exchange traits, and damages photosynthetic pigments, photosynthetic machinery, membrane integrity, photophosphorylation, and photoassimilate translocation that also triggers secondary oxidative stress (Asim et al. 2021). Plant internal water contents are the most critical variable in response to changes in temperature. Generally, plants tend to maintain balanced tissue water status for normal growth. However, HS increases the transpiration rate, therefore decreasing the water contents that directly disturb the physiology of plants (Demirel et al. 2020). In tomatoes, HS disturbed the leaf water contents and hydraulic conductivity of the roots. During the daytime, the transpiration rate increases water deficiency in plants, causing a reduction in water potential (Raja et al. 2020).

Photosynthesis is an important physiological process for carbon cycling. As it largely determines the growth and productivity of vegetables. However, under HS, leaves trap higher light energy that damages the photosynthetic machinery and reduces the photosynthetic rate (Abdalla et al. 2020). It is considered as the first sensor of environmental stress that initiates imbalanced cellular energy as observed in the altered redox chemistry related to the thylakoid membrane. It is highly sensitive to HS as it immediately restricts gaseous exchange compared with the impairment of other cellular functioning (Yalçin and Öztürk Gökçe 2021). Stomatal closure is mainly responsible for decreased photosynthetic rate as it restricts the exchange of gases after exposure to HS. Both stomatal conductance and photosynthetic rate are restricted due to moderate HS in many vegetables with the reduction in activation of rubisco (Asim et al. 2021; Haque et al. 2021). Temperature change not only affects vapor pressure but can also change the plant hydraulic conductance resulting in decreased supply of water to the leaf (Grossiord et al. 2020). Therefore, it also disrupts the transpiration rate. The plant’s capacity for sustaining the exchange of leaf gas and the assimilation rate of CO_2_ is directly associated with heat tolerance. HS affects the leaf water status, stomatal conductance, and intracellular concentration of CO_2_. In cabbage, the photosynthetic rate decreased dramatically due to the deactivation of Rubisco which is attributed to the adverse effects of heat on the electron transport system (Lee et al. 2021). Several reports have highlighted that HS caused a reduction in chlorophyll contents by impairing the biosynthesis of chlorophyll. It is attributed to the destruction of numerous enzymes involved in chlorophyll synthesis. For instance, the first enzyme, 5-aminolevulinate dehydratase, involved in the pyrrole biosynthetic pathway was reported to decrease in response to HS (Fahad et al. 2017). Protochlorophyllide (Pchlide) synthesis and Pchlide oxidoreductase are analogously decreasing in plants. It might be the reason for the reduction in chlorophyll synthesis of the vegetables. Cauliflower exposed to HS decreased the fluorescence, chlorophyll levels, and photosystem performance due to damage to photosynthetic apparatus (Rurek et al. 2018). Tomato plants acclimatized to 32 ℃ showed decreased net CO_2_ uptake accompanied by a reduction in stomatal conductance, which suggested the sensitivity of tomatoes to a temperature above 30℃. Additionally, it also affected the fruit quality and discoloration of tomatoes (Rodriguez-Ortega et al. 2017). The reduction of photosynthesis rate in tomatoes is due to decreased chlorophyll contents under HS (Zhou et al. 2017). Higher respiration rate due to high night temperature reduced the sugar contents of the peas and the marketability of the product. Gradual exposure of potato to HS decreases the internal water contents, damages the photosynthetic pigments, and decreases the gaseous exchange traits (Şanlı and Öztürk Gökçe 2021). It was noticed that photosynthesis in plants is also affected by HS due to the heat sensitivity of Rubisco and Rubisco activate enzyme. In tomato, HS at 40 ℃ reduces the addition of Rubisco enzyme isoforms (Parrotta et al. 2020). A similar decrease was also observed in pea (Haldimann and Feller 2005), spinach (Zhao et al. 2018a, b) and potato (Cen and Sage 2005).

### Production of ROS in response to stress

**Fig. 3:**
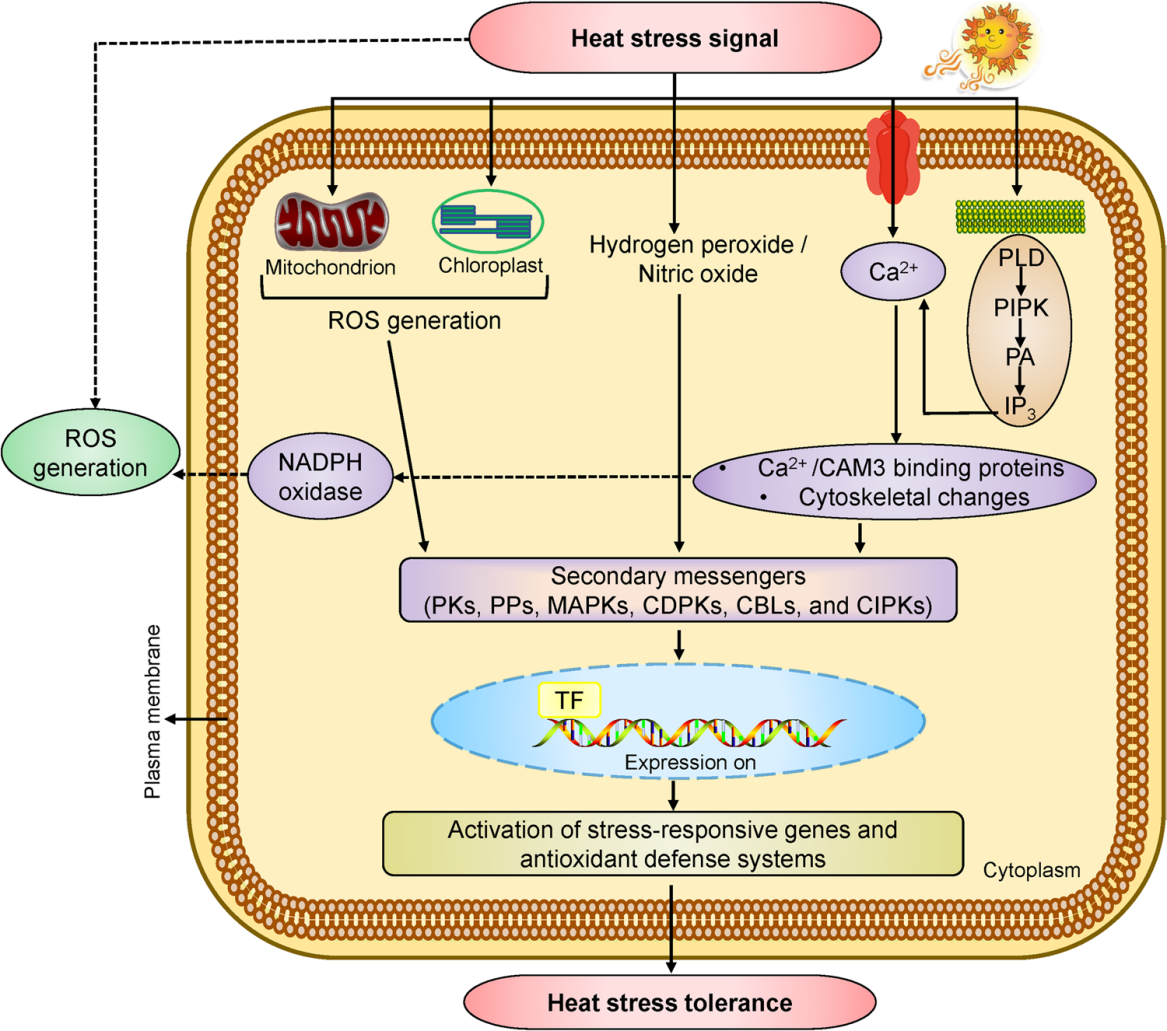
Different signaling and defense pathways in response to heat stress. Heat stress affects the plasma membrane to stimulate Ca^2+^ channels, inducing the influx of Ca^2+^. Consequently, the cascade of secondary messengers leads towards the activation of TF and gene expression. Secondary signals like hydrogen peroxide, nitric oxide, ROS, and antioxidant defense systems lead to HS tolerance. ROS: reactive oxygen species; Ca^2+^: calcium ions; PKs: ROS-induced protein kinases; PPs: protein phosphatases; MAPKs: mitogen-activated protein kinases; CDPKs: calcium-dependent protein kinases; CBLs: calcineurin-B-like proteins; CIPKs: CBL-interacting protein kinases; CAM3: calmodulin; PLD: phospholipase D; PIPK: phosphadidylinositol-4,5-biphosphate kinase; PA: phosphatidicacid; IP3: D-myo-inositol-1,4,5-triphosphate; DAG: diacylglycerol; NADPH: nicotinamide adenine dinucleotide phosphate; TF: transcription factor (such as heat shock TFs)

Reactive oxygen species (ROS) are the by-products of aerobic respiration required for the normal growth of plants because these species are directly involved in very vital physiological phenomena occurring in plants like; seed germination, growth of pollen tubes, and root hair cell expansion (Hasanuzzaman et al. 2020; Mittler et al. 2022). In response to HS, ROS are directly produced in plants (as highlighted in Fig. 3). Nevertheless, if these molecules surpass the threshold quantity and start to accumulate in various organelles, they can cause damage to the cell. Recent studies have shown that these ROS molecules are also produced in response to various abiotic stresses (Das and Roychoudhury 2014; Mittler et al. 2022). ROS family includes hydroxy radicles, hydrogen peroxide, singlet oxygen, and superoxide radicles. Among all the ROS, hydroxy radicles are considered most toxic because they rapidly react with almost all biomolecules. It is produced and accumulates in cellular membranes, chloroplast, and mitochondria, ultimately trigger cell death (Meitha et al. 2020). Hydrogen peroxide is relatively less toxic than other ROS. It is generally produced in plants because of oxidative stresses, which occur due to wounding, cold temperature, drought, light, and UV stress. It is produced by electron transport chains, cell membranes, oxidation of lipid molecules, and photorespiration. High concentrations can cause programmed cell death and enzymatic damage (Asaeda et al. 2020). Singlet oxygen is also produced because of abiotic stresses and can cause damage to lipids, proteins pigments, and nucleic acids. Photosystems of plants are also directly affected by these ROS species (Czarnocka and Karpiński 2018; Mittler et al. 2022). Superoxide radicles are moderately reactive because they are not so toxic; instead, they can cause severe membrane damage (Anjum et al. 2020).

In plants, ROS are mainly produced in chloroplasts and mitochondria. The endoplasmic reticulum, peroxisomes, and plasma membranes are also chief sites to produce these species. In chloroplasts, thylakoid membranes are involved in ROS production because these are the main sites for harvesting light. Photosystems are the sites where ROS generation occurs due to the occurrence of electron seepage, producing superoxide radicles and singlet oxygen (Sharma et al. 2020; Mittler et al. 2022). Singlet oxygen causes the peroxidation of lipid molecules of membranes which causes permanent damage to the cell and eventually causes cell death. It can also cause growth inhibition in plants (Verma et al. 2019). Mitochondria are the production house of the most toxic species of ROS. The mitochondrial electron transport chain provides energized electrons which reduce oxygen molecules to reactive oxygen species (Gautam et al. 2017). Mitochondrial enzymes like 1-galactono-γ-lactone dehydrogenase (GAL), APX, and SOD are also involved in the production (García-Caparrós et al. 2020). Mitochondrial ROS are mainly produced in drought stress conditions because the rate of respiration is reduced, which lowers the rate of ATP production in the chloroplast. To compensate for the loss of ATP, mitochondrial ATP is generated at higher rates which eventually increases ROS production (van Aken 2021). In peroxisomes, ROS generation is at higher rates due to the occurrence of integral oxidative metabolism. Xanthine oxidases present in the peroxisomal matrix and NADPH-dependent electron transport chain are the major sites for ROS production in peroxisomes. During HS, when stomata close, the rate of photorespiration increases, which increases the amount of glycolate. This glycolate is then oxidized to produce hydrogen peroxide (Qi et al. 2018). In plasma membranes, the presence of NADPH-dependent–oxidases transfers electrons to oxygen molecules which then dismutase to hydrogen peroxide, while in the apoplast, which is the space surrounding the plasma membrane, apart from NADPH oxidases, pH-dependent peroxidases (POXs), cell wall-linked oxidases, germin-like oxalate oxidases, and polyamine oxidases are responsible for the ROS production (Haider et al. 2021). In plant cell walls, during stress conditions, polyunsaturated fatty acids such as hydroperoxide convert to hydroxyl ions, singlet oxygen, and hydrogen peroxide by the activity of lipoxygenase (Janku et al. 2019). HS causes increased ROS production by affecting mitochondrial function, which results in increased peroxidation of lipids due to oxidative damage. Thus, HS causes oxidative damage to cells, sometimes beyond repair, due to uncontrolled ROS production (Qamer et al. 2021). The following section discusses the recent research on ROS production under HS, and some examples are also highlighted in Table 2.

**Table 2:** ROS production in vegetables under heat stress

Vegetable	Temperature	ROS production	References
Radish	40 ℃	H_2_O_2_	Chen et al. (2014)
Cucumber	40 ℃	O_2_•− and H_2_O_2_	Li et al. (2016)
Tomato	39 ℃	H_2_O_2_	Sakhonwasee and Phingkasan (2017)
Pepper	45 ℃	O_2_•− and H_2_O_2_	Feng et al. (2019)
Potato	37 ℃	O_2_•− and H_2_O_2_	Xi et al. (2020)
Water Spinach	42 ℃	O_2_•− and H_2_O_2_	Guo et al. (2020)
Garlic	35 and 45 ℃	H_2_O_2_	Ji et al. (2021)
Eggplant	45 ℃	H_2_O_2_	Hannachi et al. (2022)
Mungbean	40 ℃	H_2_O_2_	Kareem et al. (2022)

### Role of antioxidant enzymes to alleviate oxidative stress

The harsh environment, abiotic stress, and sessile nature of plants have given plants coping mechanisms to fight against oxidative damage caused by ROS (Hasanuzzaman et al. 2020; Raza et al. 2022d; Mittler et al. 2022). The antioxidant mechanism of plants is generally divided into two categories, i.e., enzymatic and non-enzymatic mechanisms. The non-enzymatic mechanism consists of ascorbic acid, GSH, α-tocopherol, carotenoids, phenolics, flavonoids, and osmolyte proline, while the enzymatic system consists of DHAR, SOD, CAT, APX, MDHAR, GPX, and GR (Hasanuzzaman et al. 2020). Here, we have discussed the enzymatic mechanisms.

Dehydroascorbate reductase is an enzyme that compensates the oxidative damage by reducing dehydroascorbate to ascorbic acid by transferring electrons from reduced glutathione. This enzyme regulates the level of ascorbic acid in both the symplast and apoplast, thus maintaining the overall redox state of plant cells (Dar et al. 2017). Glutathione reductase is also an important antioxidant enzyme that belongs to the flavoprotein reductase family of enzymes that produce reduced glutathione. This enzyme is mainly found in cytosol and mitochondria. It minimizes the oxidation of thiol groups, and scavenges the singlet oxygen and hydroxyl ions (Hasanuzzaman et al. 2019). Heme containing guaiacol peroxidase reduces the amount of hydrogen peroxide during stress conditions in plants by degrading indole acetic acid. It is predominantly present in cytosol, cell wall, and vacuoles (Kidwai et al. 2020). APX regulates the amount of hydrogen peroxide in peroxisomes. It uses ascorbic acid as a reducing agent to reduce hydrogen peroxide. It presents in cytosol, mitochondria, chloroplast, and peroxisomes. During stress conditions, it is considered an excellent source of scavenging H_2_O_2_ (Dumanović et al. 2021). MDHAR is another important antioxidant enzyme that is mostly present in chloroplast, mitochondria, peroxisomes, glyoxysomes, and cytosol, where it regenerates ascorbic acid using NADPH as a reducing agent. Thus it replenishes the pool of ascorbic acid in the cell (Banerjee and Roychoudhury 2017).

SOD is another important enzyme that protects the plant from oxidative damage. It is known as the first line of defense against ROS. SOD ions into an oxygen molecule and H_2_O_2_, which lowers the chances of production of hydroxyl ions. SOD has an affinity to bind with three important metal ions, i.e., Fe, Mn, and Cu. Manganese bound SOD is present in mitochondria, iron-bound SOD is present in chloroplast, while copper bound SOD is preset in cytosol and peroxisomes. Moreover, stress conditions upregulate the SOD production (del Río et al. 2018; Su et al. 2021). Catalase is another crucial antioxidant enzyme that mitigates oxidative damage by scavenging hydrogen peroxide and convert into water and oxygen. The key aspect of this enzyme that it does not require any cellular reductant to dismutase reactions. It is abundantly present in cytosol and mitochondria and protects the thiol species from oxidative damage (Ali et al. 2020; Raza et al. 2021c; Mittler et al. 2022).

### Role of osmolytes as plant abiotic stress respondents

**Fig. 4:**
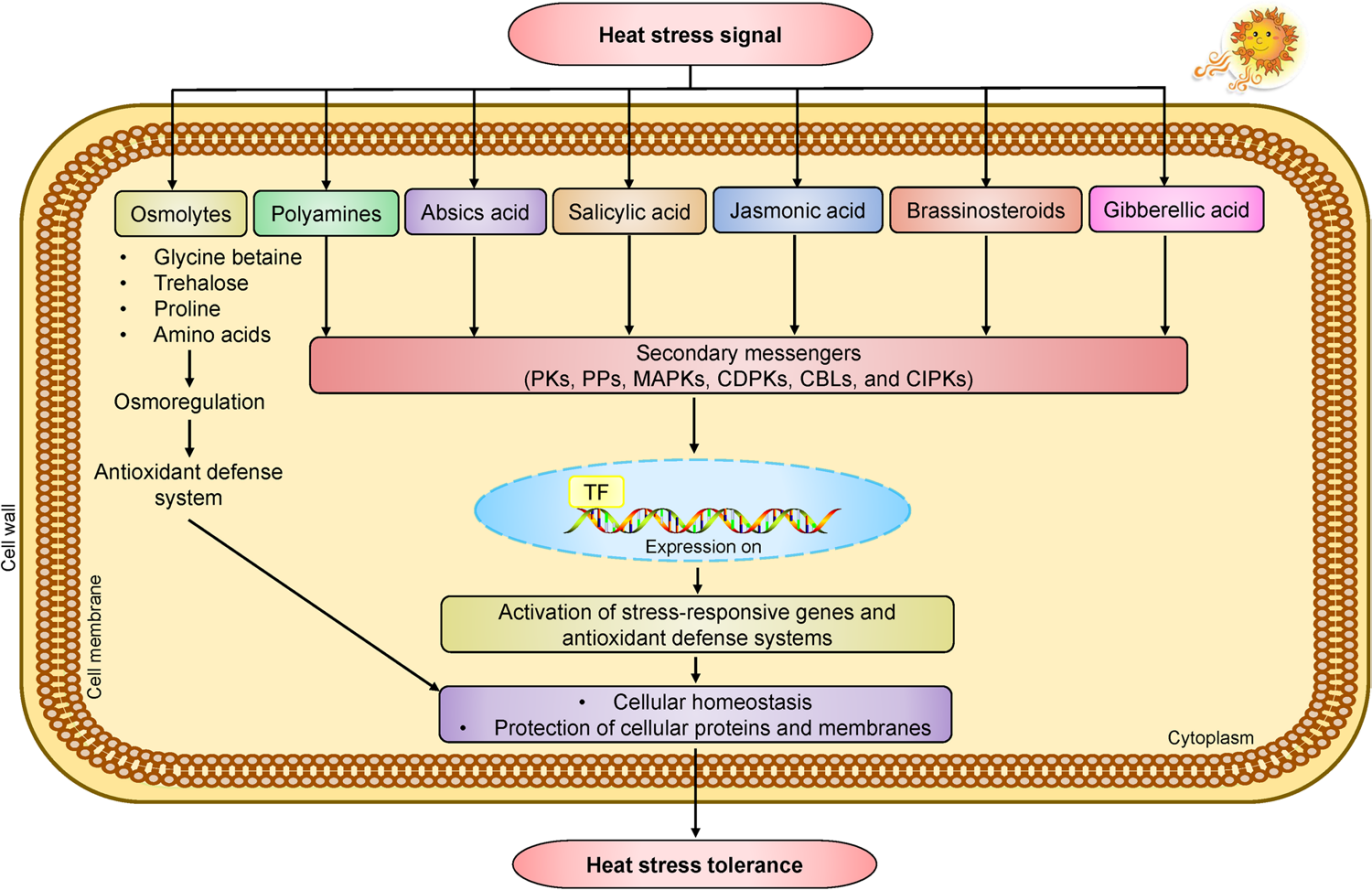
Schematic layout illustrating the participation of several osmolytes, polyamines, and phytohormones under heat stress. Compatible osmolytes such as proline, glycine betaine, and trehalose alleviate stress by osmoregulation and improving the activities of antioxidant defense systems. Different osmolytes, polyamines, and phytohormones activate the secondary messenger’s cascade leading to alteration of TFs, resulting in the activation of stress-responsive genes and antioxidant enzymes, providing stress tolerance by maintaining cellular homeostasis and protecting the cellular proteins and membranes. PKs: ROS-induced protein kinases; PPs: protein phosphatases; MAPKs: mitogen-activated protein kinases; CDPKs: calcium-dependent protein kinases; CBLs: calcineurin-B-like proteins; CIPKs: CBL-interacting protein kinases; TF: transcription factor (such as heat shock TFs)

During abiotic stress, osmolytes act as a major defensive strategy to combat ROS production. Phytohormones and osmolytes are low molecular weight metabolites (amino acids, polyols, polyamines, and sugars) that act as regulators of homeostasis and also known as cytoprotectants because they also protect cells from abiotic stress (Fig. 4) (Khan and Shahwar 2020; Raza et al. 2021a, 2022d). Osmolytes mitigate the adverse effects of abiotic stresses by protecting the molecular membrane structure and scavenging ROS. Various signaling molecules which are produced at the time of stress trigger the biosynthesis and accumulation of osmolytes (Jogawat 2019). Here we will discuss the production and role of different osmolytes in response to different plant stresses, including temperature. Proline is considered as the most important osmolyte to protect the plant against abiotic stress, and it acts as an antioxidant, molecular chaperon and glutamate as the precursor for its synthesis (Sharma et al. 2019). It has been reported that proline defends the plant against drought and HS in tobacco plants (Fahad et al. 2017). Moreover, the effect of proline as a defendant against different stresses in pea (Shahid et al. 2014), tomato (Kahlaoui et al. 2018), lentil (Molla et al. 2014), and common bean is also documented (Mahmud et al. 2020). Glycine betaine is also important for osmoregulation in the plant, which also plays an important role in protecting the plant against heat, drought, and salinity. It helps in retaining membrane structure and photosynthetic machinery, preventing ROS accumulation (Singh and Thakur 2018). Choline and glycine are the precursor molecules for the production of glycine betaine (Siddiqui et al. 2021). Polyols which are sugar alcohols, also important osmolytes. Sorbitol, inositol, mannitol’s, pinitols, and ononitols are an example of polyols that protect the plant from oxidative damage and scavenging ROS (Kumar Ghosh et al. 2021). Polyols protect the plant against CI and CS (Sanches et al. 2021).

Carbohydrates and soluble sugars are considered as important osmolytes as well because they provide osmotic balance during abiotic stresses by stabilizing the protein structure of membranes. The sugar formation from glucogenesis (noncarbohydrate precursors) is vital for plants (Zulfiqar et al. 2020). It is reported that sucrose and trehalose provide drought stress tolerance to *Craterostigma plantagineum* (Ozturk et al. 2021). Raffinose protects against extreme CS and drought in Arabidopsis (Li et al. 2020). Plants also accumulate various types of sugars to protect themselves from abiotic stress (Raza et al. 2022a, c, d; Raza et al. 2021a; Pattnaik et al. 2021).

## The interplay of polyamines and temperature in vegetables

Polyamines (PAs) are known as simple molecules which are present abundantly in vegetables. They are simple in structure. There are two types of polyamines: simple PAs like diamine putrescine (Put), cadaverine (Cad), Spermidine (Spd), and higher PAs like triamine spermidine (Spr). These PAs are present constantly in all vegetable crops and play their part in different physiological and developmental processes (Bano et al. 2020). Whenever plants face temperature stress, PAs help in increasing photosynthesis, antioxidant capacity, and osmotic adjustment, which helps to make the plant tolerant against HS (Fig. 4). PAs can be involved in various functions but are very important in different kinds of physiological mechanisms of HS tolerance (Shao et al. 2015). In the below section, we discuss the potential of PAs in mitigating the adverse effect of temperature stress.

All vegetables have an optimum temperature range where all physiological and other functions occur normally. Temperatures above or below that range can be harmful and ultimately affect the plant’s growth, development, and production. For example, when fennel seeds were treated with PAs the germination and growth of plants remarkably increased under low temperatures as compared to non-treated seeds (Mustafavi et al. 2015). In tomato seeds, PAs treatment increased the chilling tolerance by reducing the MDA level (Song et al. 2014). Several studies are documented supporting the treatment with PAs; when applied exogenously, it increases the tolerance of plants under stress (Todorova et al. 2015). In vegetables, thermotolerance could be achieved by overexpressing yeast gene SAMDC in plants. This gene caused an increased level of several PAs (Cheng et al. 2009). Cheng and colleagues studied expression profiles of tomato plants under HS treatment and applied PAs. Under Spd application, the genes expressed differently as compared to under stress conditions without PAs. The expression level of various genes involved in signaling increased when Spd was applied to plants. It shows that Spd plays a significant role in tomato plants during temperature stress (Cheng et al. 2012). So, it indicates that using different approaches to increase the level of PAs can be helpful to make plants tolerant to HS or CS (Fig. 4).

A recent study explored the effects of exogenous arginine application on the chemical and physical quality of *Brassica oleracea* under HS. The findings revealed that 1 and 4 mM of arginine application improved the activities of antioxidant enzymes, proteins, polyamines, and total phenolic compounds. The results indicated that the combination of appropriate foliar arginine treatment under HS might be a suitable strategy to raise the number of valuable plant compounds in our diet (Collado-González et al. 2021a). In another study, the physiological mechanism of exogenous spermidine on *Cucumis sativus* L., grown at high-temperature stress (42/32 °C) and treated with 1.0 mmol L^−1^ of spermidine was investigated. The results showed that exogenous Spd relieved the photosynthetic damage caused by HS, maintained the chloroplast structures, and increased chlorophyll content. Moreover, Spd reduced HS-induced photosynthesis damage by improving thylakoid membrane proteins’ expression and synthesis, alleviating the dissociation of thylakoid membrane protein complexes and LHCII–Chl, and retaining the functional stability and integrity of the photosynthetic organ structure (Wang et al. 2018a). HS reduced the soluble sugars, but exogenous Spd treatment (4 mM) improved the contents of sucrose, fructose, inositol and the bioactive nutritional constituents of *Brassica oleracea*. Furthermore, foliar application of Spd caused an increase in the antioxidant capacity and reduced, the Na^+^ and Cl^−^ ions accumulation under HS (Collado-González et al. 2021b). High-temperature stress (35℃/30℃) inhibited the lettuce seedling growth, reduced the chlorophyll a and b contents by 27.78% and 28.57% than control, the transformation from porphobilinogen (PBG) to uroporphyrinogen III (urogen III) was promoted, consequently blocking chlorophyll synthesis. Spraying 1 mM of exogenous Spd on the lettuce seedlings improved the chlorophyll a and b contents, reduced the transformation process from PBG to urogen III, and inhibited the excess protoporphyrin IX (Proto IX) accumulation, avoiding oxidative damage in the chloroplast. Exogenous Spd effectively relieves damage to lettuce at HS (Yang et al. 2022). HS had a positive effect on the quality of melon fruits; meanwhile, it improved the polyamines contents and antioxidant capacity, and total sugars and decreased the presence of unwanted substances in foods like nitrate. But, the fruit quality was increased further by the mixture of HS and putrescine (5 mmol L^−1^). In this case, the melon fruits increased their antioxidant capacity and polyamines contents, amino acids, and minerals valuable to health. This study highpoints the likelihood of refining the nutritional quality of melon pulp by applying the foliar putrescine in arrangement for a short period of HS (Piñero et al. 2021). In a study, the authors examined the effect of the exogenous putrescine application together with the different ratios of nitrate/ammonium (NO_3_^−^/NH_4_^+^) application on the cauliflower physiology subjected to HS. The 50:50 NO_3_^−^/NH_4_^+^ ratio was the best against HS. These findings revealed that the joint application resulted in a higher photosynthetic rate and a higher accumulation of photosynthesis-related compounds, pigments, and total proteins. The combined effect also stimulates the calcium, chloride, and sulfate contents in plants under HS (Collado-González et al. 2021c).

## Sensing and signaling of stress

Due to global warming and day-by-day increasing temperature, plants must evolve and adapt to the changing environment to survive. Plants have evolved various physiological pathways and cellular signaling cascades in response to these stresses, and these aspects are well studied. Diverse types of signal transduction pathways and defense mechanisms are involved in sensing ROS and effectively delivering HS-smart vegetable crops (Fig. 3; Fig. 4). However, the mechanism of signal perception in plants from outside environments still needs further study. Sensory mechanisms that convert the physical and chemical signals from the environment into biological signals need to be explored (Imran et al. 2021). Thermo sensing plays a vital role in plant stress sensing mechanism because HS not only changes the overall physiology of plant but also brings about changes in DNA, RNA, enzyme kinetics, membrane fluidity, protein structure, and folding; thus, systemic detection of these alterations is required to draw an elaborate picture of plant HS sensing mechanisms and downstream signaling cascades (Vu et al. 2019).

In plants, generally, two types of temperature sensing mechanisms are present, (1) for mild fluctuations in ambient temperature; the phytochrome system, and (2) *HSP* for HS. Normal growth and development of plants are dependent upon slight changes in ambient temperature, and it is referred to as thermo-morphogenesis, which includes the elongation of roots, hypocotyls, and petioles. The change in ambient temperature is sensed by the photoreceptors such as Phy B, which is also involved in light-sensing (Delker et al. 2014). Phy B exists in two states: pfr (far-red absorbing) and pr (red absorbing). Reversion of these states occurs spontaneously in response to HS. Thermal reversion is a key characteristic of the phytochromes; intramolecular and intermolecular interactions can regulate it. This thermo-reversion of Phy B is involved in the negative regulation of *PIF4*. Since *PIF4* controls thermogenesis*,* thus HS promotes cell development by inactivating Phy-B, which results in the accumulation of *PIF4* (Legris et al. 2016; Bellstaedt et al. 2019; Lamers et al. 2020). When the temperature increases, it increases the rate of transpiration, which results in the loss of water from the plant body.

Moreover, HS disrupts the activity of enzymes and results in misfolding and denaturation of proteins which are sensed by *HSPs*. HS and increased transpiration result in the denaturation of water-soluble proteins, thus exposing the core region of these proteins, which are most often hydrophobic (Fragkostefanakis et al. 2015). Since hydrophobic regions tend to attract and bind with other hydrophobic regions of other proteins thus, a cluster of proteins is formed. *HSP* binds to these proteins and releases Heat shock factors which bind with the heat shock elements, which regulate the transcription. *HSPs* also act as molecular chaperones and regulate the misfolded proteins (Jacob et al. 2017). Thus, HS upregulates the production of *HSPs*. In this way, *HSPs* act as signaling molecules for heat shock.

## Role of plant heat-shock transcriptional factors

*HSFs* are a complex network of a transcriptional regulatory system that controls the downstream responses in plants during heat shock and other abiotic stress responses. Its complex role in plants is highlighted in recent studies (Ohama et al. 2017; Haider et al. 2022). *HSFs* complex comprises 21 factors which are further subdivided into three families; a, b and c; their role is extensively studied in tomato (Khan and Shahwar 2020) and *Arabidopsis* (Friedrich et al. 2021). *HSFA1* subfamily acts as a master regulator of thermotolerance in tomatoes during HS (Andrási et al. 2021). Phylogenetic analyses and genome-wide studies revealed that potato *StHSFs* comprised of 27 members, which are further categorized into three groups: a, b, and c. These genes are directly involved in heat shock and other abiotic stress responses. *StHSF004*, *StHSF007*, *StHSF014*, and *StHSF19* have constitutive expressions and are expressed in both stress and non-stress conditions and thus proved as important regulatory factors during heat shock stress (Tang et al. 2016). *HSFs* in peppers (*Capsicum annum*) are not widely studied yet. However, a recent study (Guo et al. 2015) revealed that the *CaHSF* family is comprised of 25 members and is also subcategorized into 3 groups. These genes are highly conserved. qRT-PCR analysis of these genes revealed that when a plant is subjected to HS (40 °C), these gene families respond, proving that *CaHSF* is highly active during heat shock stress. *HSFs* in eggplant (*Solanum melongena* L.) comprise 20 members and are subcategorized into 14 groups. These factors not only respond to heat shock, but qRT-PCR has revealed that these *HSFs* responded to cold, salinity, and drought stress (Wang et al. 2020). Similarly, in carrot (*Daucus carota*)*,* 35 *HSFs* were identified, and these factors responded to heat shock and other abiotic stresses (Huang et al. 2015).

Apart from *HSFs* other transcriptional factors also play a key role in the thermotolerance of plants. Another important transcriptional factor is *DREB*. These transcriptional factors bind to *DRE/CRT* elements and regulate the plant response in case of HS (Singh and Chandra 2021). *DREB* factors are divided into six subgroups, i.e., *A1*, *A2*, *A3*, *A4*, *A5*, and *A6* or four classes (I, II, III, and IV) (Wang et al. 2019b). The role of *DREBA* in response to cold and drought was first studied in *Arabidopsis*. *DREB* factor not only regulates cold and drought response but is also responsible for heat tolerance in plants (Sharma et al. 2021). A recent study has revealed that *DREB* transcription factor *SlDREBA4* is responsible for improved thermotolerance in tomatoes by regulating the downstream cascade of *HSP* (Mao et al. 2020). *DREB* factors upregulate the expression of *HSPs* by acting as a transcriptional activator of *HSFA3* during heat response (Sun et al. 2020). Apart from *HSFs* and *DREBA* recently, two new genes have been reported, i.e., *Heat InducedTAS1 Target1* (*HITT1*) and *HITT2,* which are involved in the thermotolerance response of the plant. When the plant is exposed to HS, these genes are highly upregulated. Moreover, overexpression of these genes enhances the expression of *HSF,* which imparts improved thermotolerance in plants. It is reported that *HITT1* acts as a cofactor of *Hsp* (Li et al. 2014). The thermotolerance effect of *HITT2* was studied in *Brassica rapa.* It was concluded that the expression of a said gene improves the survival chances in heat shock by increasing the hypocotyl length and decreasing the electrical conductivity (Jiang et al. 2018).

## Temperature stress management using genetic approaches

Abiotic stress, mainly CS and HS, affects the production of all major vegetables worldwide. Conventional breeding strategies have not been efficient in solving the concerns of abiotic stress. May be the major reason is that these traits are controlled by more than one gene. It is important to find a candidate gene that is associated with temperature stress to produce a resilient crop. This abiotic stress affects the growth and vegetative part, directly affecting production (Parmar et al. 2017). Temperature-resilient crops can be produced for the future by profoundly investigating the relationship between genotype and phenotype. There is a need to understand variations when crops are germinated or cultivated under controlled or field conditions (normal and stressed).

### Role of QTLs and GWAS

Plant responses under HS applications can be easily detected by using different approaches such as GWAS and QTL. These approaches help to understand phenotypic traits linked with different genetic variations under different climate conditions. In this century, rapid progress in the field of biotechnology and molecular biology help researchers to understand molecular signals in the most precise manner. The progress in NGS techniques and use in GWAS help researchers find accurate genetic signals and different pathways, which ultimately help produce resilient crops against extreme temperatures (Varshney et al. 2014, 2019). GWAS and QTL can also be combined by using expression profiling based on NGS (eQTL) or transcriptome-wide association studies (TWAS). The candidate genes which are related to the phenotype of interest can be identified using these modern approaches (Nguyen et al. 2019). Some studies identified vital QTL and GWAS loci in crops linked with tolerance against stress (Kole et al. 2015). Tomato is highly affected by HS, which gradually affects the yield and quality of fruit. To overcome this issue, it is necessary to find out temperature stress-responsive genes which can be used for further breeding programs. By this method, it is easy to produce temperature-tolerant crops. There are few reports on fine mapping of HS-related QTLs and stress-related genes in tomatoes. Wen and colleagues recently performed experiments in tomato crops, showing QTL analysis of stress-related genes against HS (Wen et al. 2019).

A study identified molecular markers in bottle gourds associated with heat tolerance. A segregating population F_2_ was established between two heat tolerant and sensitive inbred lines. The population was phenotyped for relative electrical conductivity (REC) upon HS treatment, used as an indicator for heat tolerance. QTL-seq was performed and identified seven heat-tolerant QTLs (*qHT1*.*1*, *qHT2*.*1*, *qHT2*.*2*, *qHT5*.*1*, *qHT6*.*1*, *qHT7*.*1,* and *qHT8*.*1*). The *qHT2*.*1* region found three non-synonymous SNPs that were potentially linked with HS tolerance. These SNPs were positioned in the genes that may have roles in signal recognition, intracellular transport, and pollen sterility (Song et al. 2020). During the reproductive stages of chickpea (*Cicer arietinum* L.), HS leads to significant yield losses. In a recent study, Jha and colleagues identified the genomic regions responsible for HS tolerance. The inbred line derived from heat-sensitive and heat-tolerant in chickpea was genotyped. Genotyping-by-sequencing (GBS) was used and assessed for two consecutive years under HS. A high-density genetic map encompassing 788 SNP markers spanning 1125 cM was assembled. Using composite interval mapping, a total of 77 QTLs (37 major and 40 minor) were found for 12 of 13 traits and thought to be involved in HS tolerance in chickpea. Moreover, 32 candidate genes in the QTL regions encode *HSP* genes (Jha et al. 2021).

Another GWAS study performed in *Medicago truncatula* identified genes or putative loci involved in regulating seed traits and their plasticity in response to HS. Chen and colleagues recognized various essential quantitative trait nucleotides and potential candidate genes involved in regulating traits under HS through post-GWAS analyses joined with transcriptomic data. Their findings revealed that *MtMIEL1*, a RING-type zinc finger family gene, is highly associated with germination speed in heat-stressed seeds (Chen et al. 2021). Moreover, a recent study reported that thermotolerance in *Cucumis sativus* L. seedlings is a quantitative trait controlled by various genes. In cucumber seedlings, Dong and colleagues detected two loci, *qHT3.1* and *qHT3.2*, and five loci, namely *qHT3.2*, *qHT3.3*, *qHT4.1*, *qHT4.2*, and *qHT6.1,* that regulate short-term extreme or long-term mild thermotolerance. Moreover, within the significant QTL *qHT3.2*, which was repetitively identified in two stress environments via two populations, candidate genes are involved in the HS response (Dong et al. 2020). Likewise, Liu and colleagues found that HS tolerance in *Cucumis sativus* L., at the adult stage, is controlled by several genes and is a quantitative trait. The loci, named *qHT1.1*, detected, and candidate genes within this QTL are involved in HS response (Liu et al. 2021b).

### New plant breeding techniques to produce temperature-resilient crops

Vegetables play an integral part in preventing major diseases and maintaining the health of human beings because vegetables contain a high amount of nutrients and phytochemicals. Due to these attributes, the consumption of vegetables should be necessary for daily diet. A recent report indicated that consumption of fruits and vegetables of more than 400 g decreases the chance of cancer and heart diseases (Aune et al. 2017). Vegetables face production losses due to HS. Plant breeders are trying to develop vegetable varieties that show resistance against this stress, but at the same time, another interlinked goal is enhanced production and improved nutrient content (Boscaiu and Fita 2020). The conventional breeding method is time consuming and laborious and can take up to between 5 to 12 years. But genome editing technologies can speed up the production of new varieties that can be useful for producing temperature-resilient vegetables and resistance against other stresses in a time period of 4 to 6 years (Ricroch 2019). Genome editing is accepted globally as new plant breeding techniques (NPBTs). It is divided into two groups, i.e., ODM and SDNs. ODMs and SDNs can both create mutations in the genome. It can play a part upregulating and downregulating the gene’s expression (Cardi et al. 2017a). In ODM, 20 to 100 nucleotides are synthetically created and transferred to plant cells using *Agrobacterium*-mediated method or particle bombardment method. In contrast, SDNs are enzymes or proteins that bind to DNA fragments ranging from 9 to 40 nucleotides. This complex of DNA bases can introduce DSBs on the target site and can alter the genome (Puchta 2017). SDNs are divided into two categories based on their discovery. Zinc finger nucleases ZFNs and TALENs are known as the first generation of genome editing tools.

CRISPR is considered the second-generation genome editing tool (Saeed et al. 2020). The researchers and private companies used SDNs frequently to make vegetables more resistant to abiotic stresses. CRISPR technology is widely used to produce desired mutations in the genome of vegetables (Adli 2018; Dangol et al. 2021). Off-target is a major concern while using these SDNs for editing. But in plants, this issue is less important because the major population of plants can be screened out, and undesired edited plants can be discarded. There is a need for improved transformation methods and bypassing the time-consuming tissue culture method (Cardi et al. 2017b). Yu et al. (2019) generated edited plants of tomatoes. They created mutations in the *MAPK3* (Mitogen-activated protein kinase 3) gene and showed increased tolerance against HS. They exhibited lower damage to a membrane having lower ROS contents. They noticed the higher activity of antioxidant enzymes and less wilting of leaves under stress (Yu et al. 2019). *Brassinazole* signaling is controlled by two TFs, known as *BZR1* and *BZR2*. Yin and colleagues knock out the tomato plants using CRISPR/cas9. They create a mutation in *BZR1,* which is known as susceptible to HS. When plants were exposed to HS, *HSP* and ROS were upregulated. However, in mutants, *HSP* and ROS were downregulated, and H_2_O_2_ production also decreased in mutants (Yin et al. 2018). Hu and colleagues generated *Solanum lycopersicum cpk28* mutants using a CRISPR/Cas9 approach. The responses of mutant and wild-type plants at 25 °C normal and 45 °C HS were documented. Thermotolerance was expressively reduced in the *cpk28* mutants revealed improved HS-induced ROS accumulation and protein oxidation levels, together with decreased APX activities and other antioxidant enzymes. So, the protein kinase *CPK28* phosphorylates the ascorbate peroxidase and enhances thermotolerance in tomatoes (Hu et al. 2021).

Genome editing applications to produce temperature or additional abiotic stresses resilient crops are so far limited to model crops. This limitation may be due to complex genetic mechanisms and signaling. Several genes are downregulated and upregulated at the same time, so to make a vegetable resilient against HS, there is a need to switch off different genes simultaneously which is more difficult than changing resistance to some biotic stresses where a single susceptibility gene knockout works (Zaidi et al. 2018). It is essential to optimize transformation protocols for major vegetables also reproducible, because obtaining homozygous stably edited plants is important. There is a prerequisite for genome editing of genes; researchers should know about genome sequences and different regions of a gene, such as an intron, exon, and promoter region, which control gene expression.

### Omics advancements in developing heat-stress resilient vegetables

Plant responses to HS relies on genes regulation (down- and up-regulation). Integrated omics research has been used in this context to understand the plant’s molecular mechanisms and biological networking against HS. Omics approaches, including transcriptomics, proteomics, and metabolomics, helps to find key genes, their interactions, and regulation developed at different metabolic pathways upon exposure to HS (Table 3) (Raza et al. 2021b; Raza et al. 2022a, b, c, d). Numerous tools have conducted transcriptomic studies, comprising a hybridization-based approach, RNA sequencing, and other sequencing applications. Transcriptomic analysis of *Ipomoea aquatica* subjected to HS of 42 °C showed that 4145 transcripts were specifically expressed. Enrichment analysis of these DEGs revealed differentially expressed genes involved in carbohydrate metabolism, phenylpropanoid biosynthesis, sugar transport, and metabolic transition (Gao et al. 2020). Transcriptome analysis of *Capsicum annuum* L. was conducted under HS (42 °C) and revealed 11,633 deferentially expressed genes (DEGs) involved in metabolic processes and photosynthesis. Moreover, 17 *HSFs*, 38 NAC (NAM, ATAF1/2, and CUC2), 35 WRKY proteins, and 38 *HSPs* were identified that were responsive to HS (Wang et al. 2021a). In another study, transcriptome analysis of *B. rapa* was performed, and a total of 11,055 and 8921 differentially expressed genes (DEGs) were identified in “268” and “334,” respectively. Functional enrichment analyses of all identified DEGs revealed that the ribosome biogenesis, autophagy pathway, and glutathione metabolism were significantly upregulated, whereas photosynthesis was downregulated in *B. rapa* “268.” In contrast, HS in *B. rapa* “334” caused the expression of specific functional genes associated with plant hormone signal transduction pathways and protein processing in the endoplasmic reticulum (Yue et al. 2021). Transcriptomic analysis of *Solanum melongena* L. at HS (45 °C) revealed the expression of genes related to anthocyanin biosynthesis (Zhang et al. 2019).

**Table 3:** Summary of key transcriptomics, proteomics, and metabolomics studies under high-temperature stress in some vegetable crops

Species	Stress condition	Specific tissue	Approach	Functional annotation method	Key findings	References
Transcriptomics						
*Capsicum annuum* L	42 °C for 3 d	Seedlings	RNA-Seq	GO, KEGG	11,633 DEGs were identified 38 heat shock factors (Hsps), 17 HS transcription factors (Hsfs), 38 NAC (NAM, ATAF1/2, and CUC2), and 35 WRKY proteins that were responsive to HS	Wang et al. (2021a)
*Capsicum annuum*	40 °C	Leaves	RNA-Seq	GO	12,494 DEGs were identified. Identified DEGs to provide various stimuli for developing HS resistant cultivars	Kang et al. (2020)
*Solanum melongena* L	38 °C and 43 °C for 3 h	Leaves	RNA-Seq	GO, KEGG	3067 and 1456 DEGs were identified. 315 and 342 genes were upregulated and downregulated DEGs involved in antioxidant enzyme systems, detoxication, phytohormones, and transcription factors	Zhang et al. (2020)
*Benincasa hispida*	45 °C/40 °C in day/night for 5 d	Leaves	RNA-Seq	GO, KEGG	1505 DEGs (914 upregulated and 591 downregulated) were identified DEGs are related to heat shock proteins (HSPs), ubiquitin-protein ligase, transcriptional factors, and pentatricopeptide repeat-containing proteins	Wang et al. (2019c)
*Solanum melongena* L	45 °C for 6 h	Fruits	RNA-Seq	GO, KEGG	770 DEGs were identified 16 genes related to anthocyanin biosynthesis	Zhang et al. (2019)
Proteomics						
*Brassica juncea*	30 °C	Sprout	Acetonitrile	LC–MS/MS, UPLC, UNIPROT, and KEGG	172 DEPs identified. Increased expression of genes/ proteins related to melatonin, electrolyte leakage, GSH, and POD. Increased defense pressure, protein biosynthesis, signal transduction, and transcription under HS. Involved in protein transport	Cheng et al. (2020)
*Brassica campestris* L	40/30 °C for 3 d	Leaf	acetate–methanol	HPLC, LC–MS/MS	1022 DEPs were identified Increased expression of genes/ proteins related to redox homeostasis, photosynthesis, carbohydrate metabolism, heat-shock protein, and chaperones and signal transduction pathways	Yuan et al. (2019)
*Spinacia oleracea* L	37/32 °C day/night	Leaves	Phenol	iTRAQ, LC–MS/MS	911 DEPs were identified related to ROS homeostasis, endomembrane trafficking, and cross-membrane transport pathways	Zhao et al. (2018a, b)
*Cucumis sativus*	42 °C for 7d	Leaves	acetone	MALDI-TOF/TOF MS, 2-DE	**77** DEPs identified involved photosynthesis, energy and metabolism, defense response, and protein and nucleic acid biosynthesis	Xu et al. (2018)
*Lactuca sativa* L	33 °C	Stems	TCA/acetone	iTRAQ, LC, ESI, MS/MS	5454 DEP identified 619 proteins induced by HS and associated with photosynthesis and tryptophan metabolism involved in IAA biosynthesis	Hao et al. (2018)
Metabolomics						
*Lactuca sativa* L	35 ◦C for 40 h	Leaves	GC–MS, UPLC-IMS-QTOF/MS	PCA	Increased accumulation of organic acids, amino acids, terpenoids, phenolic compounds, carbohydrates, and lipids are involved in lettuce seed germination and thermo-inhibition	Wei et al. (2020)
*Sargassum fusiforme*	32 °C for 7 d	Leaves	GC–MS	OPLS-DA, PCA, KEGG	A heat shock increases the production of organic acids, amino acids, sugars or sugar alcohols, esters, and amines Changes in metabolic pathways may contribute to the HS tolerance	Liu and Lin (2020)
*Capsicum annuum* L	~ 40 °C for 28 h	Leaves	LC–ESI–MS/MS, ESI-Q TRAP-MS/MS	PLS-DA, PCA, KEGG	94 and 108 differentially accumulated metabolites (DAMs) were identified Amino acids, organic acids, flavonoids, and sugars are involved in HS tolerance	Wang et al. (2019a)
*Solanum lycopersicum*	40 °C/30 °C (day/night) over a 48 h	Fruits	GC–MS	PCA, UPLC-PDA	Increased accumulation of threonine and β-sitosterol levels. Proline and GABA responsive to HS	Almeida et al. (2021)
*Solanum lycopersicum*	38 °C for 1 h	Leaves	GC-TOF–MS, LC-QTOF-MS	PCA	Increased accumulation of sucrose, glucose, putrescine, and caffeoyl quinic acid isomers involved in HS tolerance	Paupière et al. (2020)

Proteomics broadly covers the proteins encoded in living organisms at a specific instance and plays a key role in understanding all cellular courses at the molecular level (Raza et al. 2021b). See Table 3 for some key examples. Using iTRAQ technology, proteome analysis of radish taproots under HS (40 °C) identified 2258 DAPs. These DAPs are mainly involved in annexin, ubiquitin-conjugating enzyme, ATP synthase, HSP, signal transduction, stress and defense pathways, photosynthesis and energy metabolic pathways, and working processes cooperatively to reduce stress-caused damage in radish (Wang et al. 2018b). In another study, Wang and colleagues conducted an iTRAQ-based quantitative proteomic analysis of pepper seedlings subjected to HS at 40 °C. In proteomic analysis, a total of 3,874 DAPs were identified, and 1,591 proteins were related to higher ROS scavenging, photosynthesis, signal transduction, carbohydrate metabolism, and stress defense (Wang et al. 2021b). In *Lactuca sativa* L., iTRAQ-based proteomics analysis was conducted. Of the 5454 identified proteins, 619 proteins showed differential abundance by HS. The proteins made under HS (33 °C) are primarily associated with photosynthesis and tryptophan metabolism involved in auxin (IAA) biosynthesis (Hao et al. 2018). A study examined heat adaptation mechanisms in *Spinacia oleracea* L. using a proteomics approach. In proteomic analysis, 911 DAPs were identified as involved in endomembrane trafficking, cross-membrane transport pathways, and ROS homeostasis (Zhao et al. 2018a, b).

Metabolomics roughly detects and quantifies all endogenous and exogenous molecules of low molecular weight, i.e., < 1 kDa, comprising metabolites in living organisms (Raza 2022). Masses of sugars (sucrose, glucose, and fructose), TCA cycle, and starch biosynthesis were strongly linked with HS tolerance (Dhatt et al. 2019). Under HS, the metabolome profile of *Lactuca sativa* L. responded differently. Heat shock accumulated higher concentrations of sugars, organic acids, amino acids, sterols, sesquiterpene lactones, and fatty acids derivatives (Wei et al. 2020). A recent study analyzed metabolic changes in the leaves of *S. fusiforme* using GC–MS under HS (32 °C). Increased electrolyte leakage and suppressed chlorophyll content were observed under HS. While, under HS (32 °C), various metabolisms expressively improved 7-pathways, including alanine, aspartate, and glutamate metabolism; aminoacyl-tRNA biosynthesis; phenylalanine metabolism; tyrosine metabolism; arginine and proline metabolism; nitrogen metabolism; and isoquinoline alkaloid biosynthesis. These metabolic pathways changes might involve the HS tolerance of *S. fusiforme* (Liu and Lin 2020). Wang and colleagues conducted an MRM mode metabolomics analysis of *Capsicum annuum* L. seedlings subjected to HS (40 °C) and identified 94 and 108 DAMs. From metabolome data, especially sugars, flavonoids, organic acids, and amino acids involved in HS tolerance in pepper (Wang et al. 2019a). Some recent examples are shown in Table 3.

A phenome is a set of biochemical, physical, and biological processes expressed by an organism in the form of phenotypes (quantitative and qualitative features) in a specific living condition. Plant phenomics illustrates the genotypic and phenotypic expression within a particular living condition (Raza et al. 2022b, c; Raza et al. 2021b). For example, a nondestructive phenomics approach was used to evaluate HS (35 °C) tolerance at anthesis in *Brassica* species. Findings reveal that flower volume was the critical phenomic character for HS tolerance in this crop. Also, whole-plant measurements were mainly related to fresh weight variations, suggesting that the entire plant imaging might be an appreciated replacement for the fresh weight in upcoming examinations (Chen et al. 2019b). By examining the relationship between the traits, results describe early differences in the photochemical quenching paralleled with the rosette extent at future steps, which advocates quenching to complete HS tolerance (Gao et al. 2020).

## Conclusions and future recommendations

Temperature stress has massively affected the growth and development of vegetables. HS (and also cold stress) directly changes the plant’s biochemical, physiological, and molecular behavior. Consequently, it will impact vegetable production. There are fewer studies regarding vegetable adaptation against HS in comparison to cereals. The conventional breeding method is used to improve vegetables against HS, but it is time-consuming and laborious. It needs precision and needs a long time to develop a tolerant cultivar. On the other side, new genomic tools are becoming available, which will reduce the time to generate tolerant HS cultivars in many vegetables. Genomics-assisted breeding (QTLs and GWAS) are less studied for producing temperature-resilient vegetable crops. The transgenic technology helped several crops to make them tolerant to HS, but research was limited to vegetable crops (chilli peppers and tomatoes). These modern genomic studies can be helpful in making other vegetables resilient against temperature. Furthermore, new plant breeding technologies can be useful in producing vegetables with our desired traits. For the success of these technologies, there is a need to understand and enhance application of these modern genomic tools. There is also a need to determine the main genetic players involved in signaling and controlling genes that play a negative or positive role during stress management. For implementing a genome editing system, the availability of vegetable genome sequences and good transformation systems are a prerequisite. The consumer’s acceptability will be more towards CRISPR-edited crops because of possible transgene-free productions of crops. There is a need to optimize genome editing technology in different vegetables, which can help produce temperature-resilient crops. In conclusion, there is a need to combine transgenic and genome-edited technologies with conventional and genomics-assisted breeding to produce temperature-tolerant crops to feed the growing global population.

## Data Availability

Not applicable.

## Abbreviations

*HS*: Heat stress
*ROS*: Reactive oxygen species
*GAL*: 1-Galactono-γ-lactone dehydrogenase
*DHAR*: Dehydroascorbate reductase
*SOD*: Superoxide dismutase
*CAT*: Catalase
*APX*: Ascorbate peroxidase
*MDHAR*: Monodehydroascorbate reductase
*GPX*: Guaiacol peroxidase
*GR*: Glutathione reductase
*H2O2*: Hydrogen peroxide
*CI*: Cold Injury
*CS*: Cold stress
*PAs*: Polyamines
*Put*: Putrescine
*Cad*: Cadaverine
*Spd*: Spermidine
*Spr*: Spermidine
*DNA*: Deoxyribonucleic acid
*RNA*: Ribonucleic acid
*HSP*: Heat shock proteins
*Phy*: Photoreceptors
*PIF4*: Phytochrome interacting factor 4
*HSFs*: Heat shock transcriptional factors
*DREB*: Dehydration responsive element binding protein
*GWAS*: Genome-wide association studies
*QTL*: Quantitative trait loci
*NGS*: Next-generation sequencing
*SNPs*: Single nucleotide polymorphisms
*ODM*: Oligonucleotide-directed mutagenesis
*SDNs*: Site-directed nucleases
*ZFNs*: Zinc finger nucleases
*TALENs*: Transcription activator-like effector nucleases
*CRISPR*: Clustered regularly interspaced short palindromic repeats
*DSBs*: Double-strand breaks
*TFs*: Transcription factors
*iTRAQ*: Isobaric tag for relative and absolute quantitation
*DAPs*: Differentially accumulated proteins
*IAA*: Indole acetic acid
*TCA*: Tricarboxylic acid
*DAMs*: Differentially accumulated metabolites
